# Establishment and validation of an extracellular volume model without blood sampling in ST-segment elevation myocardial infarction patients

**DOI:** 10.1093/ehjimp/qyae053

**Published:** 2024-06-10

**Authors:** Lei Chen, Zeqing Zhang, Xinjia Du, Jiahua Liu, Zhongxiao Liu, Wensu Chen, Wenliang Che

**Affiliations:** Department of Cardiology, Shanghai Tenth People’s Hospital, Tongji University School of Medicine, 301 Yanchang Road, Shanghai 200072, China; Department of Cardiology, The Affiliated Hospital of Xuzhou Medical University, 99 Huaihai West Road, Xuzhou 221002, China; Department of Cardiology, The Affiliated Hospital of Xuzhou Medical University, 99 Huaihai West Road, Xuzhou 221002, China; Department of Cardiology, The Affiliated Hospital of Xuzhou Medical University, 99 Huaihai West Road, Xuzhou 221002, China; Department of Radiology, The Affiliated Hospital of Xuzhou Medical University, 99 Huaihai West Road, Xuzhou 221002, China; Department of Cardiology, The Affiliated Hospital of Xuzhou Medical University, 99 Huaihai West Road, Xuzhou 221002, China; Department of Cardiology, Shanghai Tenth People’s Hospital, Tongji University School of Medicine, 301 Yanchang Road, Shanghai 200072, China

**Keywords:** cardiac magnetic resonance, extracellular volume, haematocrit, synthetic, T1 mapping

## Abstract

**Aims:**

Recent studies have shown that extracellular volume (ECV) can also be obtained without blood sampling by the linear relationship between haematocrit (HCT) and blood pool R1 (1/T1). However, whether this relationship holds for patients with myocardial infarction is still unclear. This study established and validated an ECV model without blood sampling in ST-segment elevation myocardial infarction (STEMI) patients.

**Methods and results:**

A total of 398 STEMI patients who underwent cardiac magnetic resonance (CMR) examination with T1 mapping and venous HCT within 24 h were retrospectively analysed. All patients were randomly divided into a derivation group and a validation group. The mean CMR scan time was 3 days after primary percutaneous coronary intervention. In the derivation group, a synthetic HCT formula was obtained by the linear regression between HCT and blood pool R1 (*R*^2^ = 0.45, *P* < 0.001). The formula was used in the validation group; the results showed high concordance and correlation between synthetic ECV and conventional ECV in integral (bias = −0.12; *R*^2^ = 0.92, *P* < 0.001), myocardial infarction site (bias = −0.23; *R*^2^ = 0.93, *P* < 0.001), and non-myocardial infarction sites (bias = −0.09; *R*^2^ = 0.94, *P* < 0.001).

**Conclusion:**

In STEMI patients, synthetic ECV without blood sampling had good consistency and correlation with conventional ECV. This study might provide a convenient and accurate method to obtain the ECV from CMR to identify myocardial fibrosis.

## Introduction

The morbidity and mortality of ST-segment elevation myocardial infarction (STEMI) have significantly improved over the decades due to the widespread use of primary percutaneous coronary intervention, the optimization of pharmacological treatments, and the standardization of secondary prevention. However, STEMI remains a leading cause of death.^1–4^ Myocardial fibrosis and structural remodelling are crucial factors contributing to a poor prognosis after myocardial infarction.^5^ Therefore, developing a more efficient assessment method for evaluating myocardial fibrosis and structural remodelling in patients with STEMI is imperative.

In recent years, T1 mapping obtained by cardiac magnetic resonance (CMR) has been widely recognized and used to assess the structural reconstruction of myocardial tissue.^6,7^ Extracellular volume (ECV), obtained by T1 mapping, enabled accurate identification and assessment of myocardial infiltration, oedema, and fibrosis, whether focal or diffuse.^8^ A previous study demonstrated a strong association between ECV and histologically determined diffuse interstitial fibrosis in valvular heart disease. It is worth noting that neither non-contrast T1 times nor the amount of late gadolinium enhancement could accurately assess the extent of diffuse interstitial fibrosis.^9^ In addition, ECV was a reliable marker for assessing major cardiovascular adverse events.^10^ Compared with T1, ECV better reflected the severity of microvascular injury and was related to poor left ventricular (LV) remodelling.^11^ Conventional ECV was calculated based on the blood pool R1 (1/T1), and it was adjusted using haematocrit (HCT) within 24 h of CMR examination,^9,12,13^ which somewhat limited the use of ECV in the clinic. Previous studies have demonstrated a linear correlation between blood pool R1 and HCT.^14,15^ However, whether this relationship holds for patients with myocardial infarction has not been reported in a topical study. Suppose we assume that there is also a linear correlation between HCT and blood pool R1 in STEMI patients. In that case, it becomes possible to synthesize ECV in these patients without blood sampling computationally. To the best of our knowledge, this is the first study to investigate synthesized ECV in an Asian population. The purpose of this study was (i) to establish a synthetic HCT model and (ii) to investigate whether synthetic HCT can be used for reliable and efficient calculation of synthetic ECV in STEMI patients.

## Methods

### Study population

**Figure 1:**
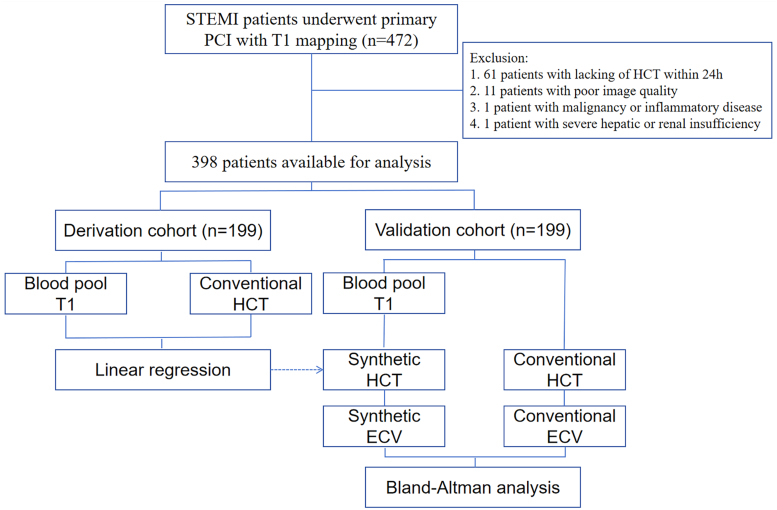
Workflow.

This retrospective study included patients diagnosed with STEMI^16^ from January 2021 to February 2023. All patients underwent CMR, which included T1 mapping sequences. The blood sampling was collected within 24 h of the CMR examination. The Institutional Review Board (IRB) approved the study protocol with the reference number XYFY2023-KL199-01. The requirement for signed written consent was waived owing to the low risk to the patient in accordance with the relevant IRB regulatory guidelines. The exclusion criteria were (i) poor image quality, including artefacts and incomplete scanning sequence; (ii) patients with severe hepatic and renal insufficiency, severe infection, or advanced tumour; and (iii) incomplete clinical data. The patients were randomly divided into derivation and validation groups (*Figure 1*). The clinical data and relevant laboratory indexes were obtained from the patient’s clinical records.

### Magnetic resonance imaging

All subjects underwent image acquisition on a 3.0T scanner (Ingenia 3.0T, Philips, Netherlands) within the first week of diagnosis of STEMI. The patients were positioned on their backs, and images were obtained using the digital stream (DS) front phased array coil and the integrated DS posterior spine matrix coil while holding their breath. An improved standard imaging protocol was used as the reference. After the injection of gadolinium-containing contrast medium (0.1 mmol/kg), short-axis images covering the LV with three to five layers were acquired, and T1 mapping (MOLLI, a modified Look-Locker) was performed before and 10–15 min after contrast agent application. The scanning parameters were as follows: slice thickness of 7 mm, echo time of 1.4 ms, complex time of 2.8 ms, visual field of 300 × 300 mm, and matrix size of 280 × 240.

### Image analysis

**Figure 2:**
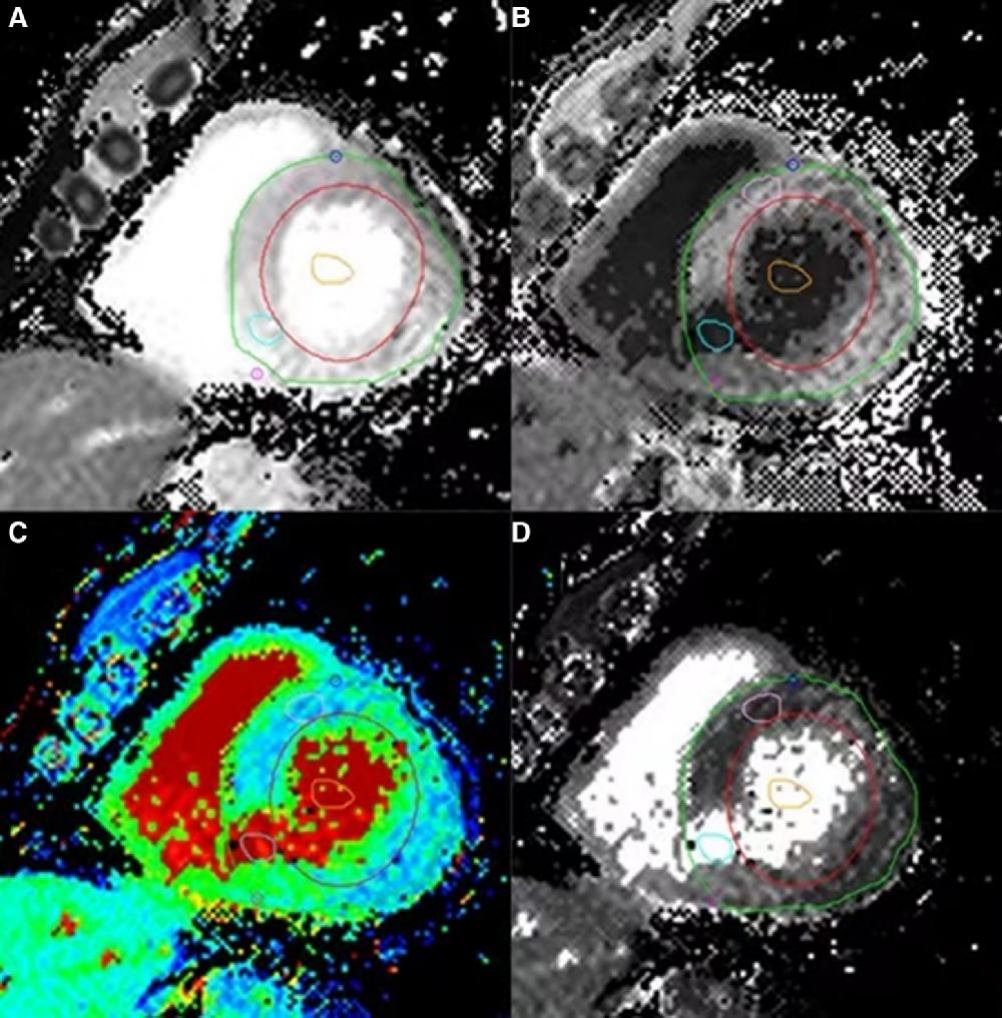
(*A*) Native T1 map; (*B*) enhanced T1 map; (*C*) generated ECV images; and (*D*) the distribution coefficient *λ* that generates the image; circles indicate different regions.

Cardiovascular imaging software CVI 42 (Circle Cardiovascular Imaging, Canada) was used to analyse the image. The endocardial, epicardial, and blood pool were delineated on a short-axis plane to assess the T1 relaxation time. Mean relaxation times (≥1 cm^2^) of two regions of interest (ROI) were plotted at the myocardial infarction site (MIS) and the non-myocardial infarction site (NMIS). Limbic and papillary muscles were carefully avoided. The T1 value of the blood pool was obtained from the LV. For the validation cohort population, routine ECV values were standardized for blood HCT and calculated using previously published equations: ECV =(1 − HCT) × (1/myocardial enhanced T1 − 1/myocardial native T1)/(1/blood pool–enhanced T1 − 1/blood pool native T1) (*Figure 2*). Two experienced radiologists, each with over 3 years of experience, analysed all CMR images. Functional parameters such as left ventricular ejection fraction (LVEF), end-diastolic volume (EDV), and end-systolic volume (ESV) were evaluated, and papillary muscles were included in the LV volume.

### Calculation method of synthetic ECV

In the derivation group, the reciprocal longitudinal relaxation time of blood (R1 = 1/T1) and HCT were analysed for their linear relationship, and a formula for synthetic HCT was obtained. The synthetic ECV was calculated using the synthetic HCT in the validation group.

### Statistical analysis

SPSS 24.0 software was used for statistical analysis. The continuous variables are expressed by mean and standard deviation, and the classified variables are expressed by percentage. Student’s *t*-test was used to analysis the continuous variables of the normal distribution, and the Wilcoxon rank-sum test was used to analysis the non-normally distributed continuous variables. The binary variable gate uses the *χ*^2^ test (count >5) and the Fisher test (count ≤5). Derivation group: using linear regression analysis to get the synthetic HCT formula. Validation group: the Bland–Altman analysis was performed for consistency between the synthetic ECV and the conventional ECV. Pearson correlation coefficient was used to evaluate the correlation between continuous variable gates. The statistical test was a two-tailed test, and *P* < 0.05 was considered statistically significant.

## Results

### Patient characteristics

A total of 398 patients were included in this study and randomly divided into a derivation group (*n* = 199) and a validation group (*n* = 199). The derivation group was used to calculate the linear regression equation of synthetic HCT and ECV. There were no statistical differences in baseline characteristics such as age and gender between the derivation and validation groups. Besides, there was no significant difference between the two groups in conventional HCT (40.81 ± 3.60% vs. 40.55 ± 4.09%, *P* = 0.491), T1 time of myocardial and blood pool (1349.49 ± 80.93 ms vs. 1349.84 ± 77.60 ms *P* = 0.965; 1802.58 ± 112.13 ms vs. 1820.33 ± 127.58 ms, *P* = 0.141), and conventional ECV in integral, NMIS, and MIS (30.45 ± 7.03% vs. 30.31 ± 5.81%, *P* = 0.831; 24.73 ± 5.63% vs. 24.05 ± 5.27%, *P* = 0.212; 46.47 ± 10.02% vs. 48.37 ± 10.38%, *P* = 0.066; *Table 1*). According to the results of CMR, there were 525 infarcts (including 2 or more infarcts) in 398 patients, as shown in *Table 2*.

**Table 1:** Patient characteristics

	All patients (*n* = 398)	Derivation (*n* = 199)	Validation (*n* = 199)	*P*-value
Age, years	56.14 ± 12.68	56.24 ± 12.90	56.04 ± 12.50	0.878
Male, *n* (%)	339 (85.18)	166 (83.42)	173 (86.93)	0.323
BSA, m^2^	1.83 ± 0.20	1.82 ± 0.19	1.83 ± 0.20	0.685
BMI, kg/m^2^	25.74 ± 3.69	25.67 ± 3.53	25.80 ± 3.86	0.726
Hb, g/L	136.74 ± 15.55	136.57 ± 15.46	136.91 ± 15.67	0.827
HCT, %	40.68 ± 3.85	40.81 ± 3.60	40.55 ± 4.09	0.491
LDL, mmol/L	2.81 ± 0.89	2.78 ± 0.90	2.84 ± 0.89	0.470
LVEF, %	54.17 ± 6.57	54.33 ± 6.04	54.01 ± 7.07	0.626
LV-edm, mm	49.89 ± 4.00	49.56 ± 3.64	50.22 ± 4.31	0.100
EDV, cm^3^	126.63 ± 32.36	123.75 ± 29.12	129.50 ± 35.14	0.076
ESV, cm^3^	59.03 ± 21.96	57.29 ± 19.54	60.78 ± 24.06	0.113
Native myo T1-time, ms	1349.16 ± 79.56	1349.49 ± 80.93	1349.84 ± 77.60	0.965
Native LV blood T1-time, ms	1811.45 ± 120.14	1802.58 ± 112.13	1820.33 ± 127.58	0.141
ECV, % (NMIS)	24.39 ± 5.46	24.73 ± 5.63	24.05 ± 5.27	0.212
ECV, % (MIS)	47.42 ± 10.33	46.47 ± 10.02	48.37 ± 10.38	0.066
ECV, % (integral)	30.38 ± 6.44	30.45 ± 7.03	30.31 ± 5.81	0.831
Peak NT-proBNP, pg/mL	1251.20 (626.80, 2194.25)	1254.00 (629.40, 2153.00)	1234.00 (614.59, 2220.00)	0.817
Peak hs-TnT, ng/L	1700 (536.55, 4674.00)	1842.00 (605.37, 4958.5)	1641.00 (506.25, 4293.75)	0.468
ACEI/ARB, *n* (%)	244 (61.31)	120 (60.30)	124 (62.31)	0.681
β-Blockers, *n* (%)	347 (87.19)	174 (87.44)	173 (86.93)	0.881
Aspirin, *n* (%)	384 (96.48)	192 (96.48)	192 (96.48)	1.000
SGLT-2, *n* (%)	107 (26.88)	56 (28.14)	51 (25.63)	0.572
P2Y12 inhibitors, *n* (%)	387 (97.24)	196 (98.49)	191 (95.98)	0.126
Statins, *n* (%)	387 (97.24)	194 (97.49)	193 (96.98)	0.760
Spironolactone, *n* (%)	28 (7.04)	15 (7.54)	13 (6.53)	0.695
Hypertension, *n* (%)	155 (38.94)	73 (36.68)	82 (41.21)	0.355
Diabetes, *n* (%)	98 (24.62)	52 (26.13)	46 (23.12)	0.485
Atrial fibrillation, *n* (%)	15 (3.77)	7 (3.52)	8 (4.02)	0.792

BSA, body surface area; BMI, body mass index; Hb, haemoglobin; LVEF, left ventricular ejection fraction; LV-edm, left ventricular end-diastolic meridian; EDV, end-diastolic volume; ESV, end-systolic volume; NMIS, non-myocardial infarction site; MIS, myocardial infarction site; NT-proBNP, N-terminal pro-B-type natriuretic peptide; Hs-TnT, high-sensitivity troponin T; ACEI, angiotensin-converting enzyme inhibitors; ARB, angiotensin receptor blocker; β-blockers, beta blockers; SGLT-2, sodium-dependent glucose transporters 2.

**Table 2:** Location of infarct in patients

	All patients (*n* = 398)	Derivation (*n* = 199)	Validation (*n* = 199)	*P*-value
Anterior, *n* (%)	174 (43.72)	88 (44.22)	86 (43.22)	0.84
Posterior, *n* (%)	21 (5.28)	10 (5.03)	11 (5.53)	0.82
Inferior, *n* (%)	66 (41.71)	82 (41.21)	84 (42.21)	0.84
Lateral, *n* (%)	86 (21.61)	43 (21.61)	43 (21.61)	1
Anteroseptal, *n* (%)	9 (2.26)	4 (2.01)	5 (2.51)	0.74
Apex, *n* (%)	34 (8.54)	21 (10.55)	13 (6.53)	0.15
Interventricular septum, *n* (%)	35 (8.79)	17 (8.54)	18 (9.05)	0.86

### Model derivation

**Figure 3:**
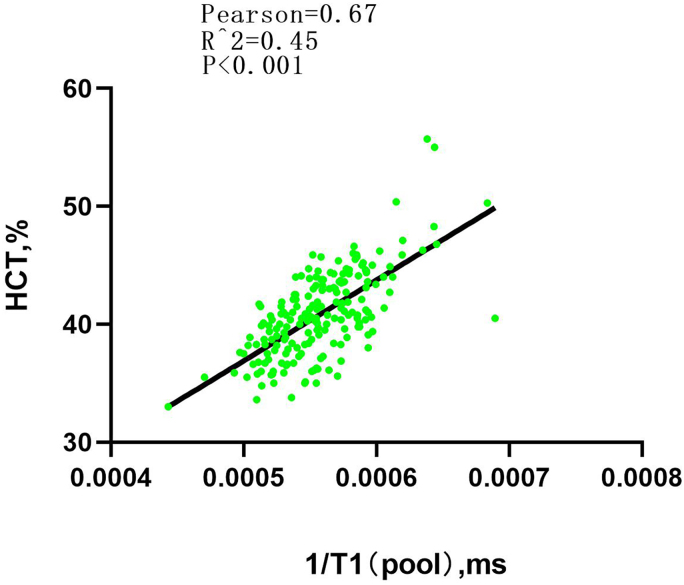
Linear regression analysis between HCT and blood pool T1.

In the derivation group, R1 and conventional HCT had a linear correlation (*R*^2^ = 0.45, *P* < 0.001). Through linear regression analysis, R1 was used to derive the formula to estimate synthetic HCT: HCT = 685.02 × (1/blood pool T1) + 0.02662 (*Figure 3*).

### Model verification

**Figure 4:**
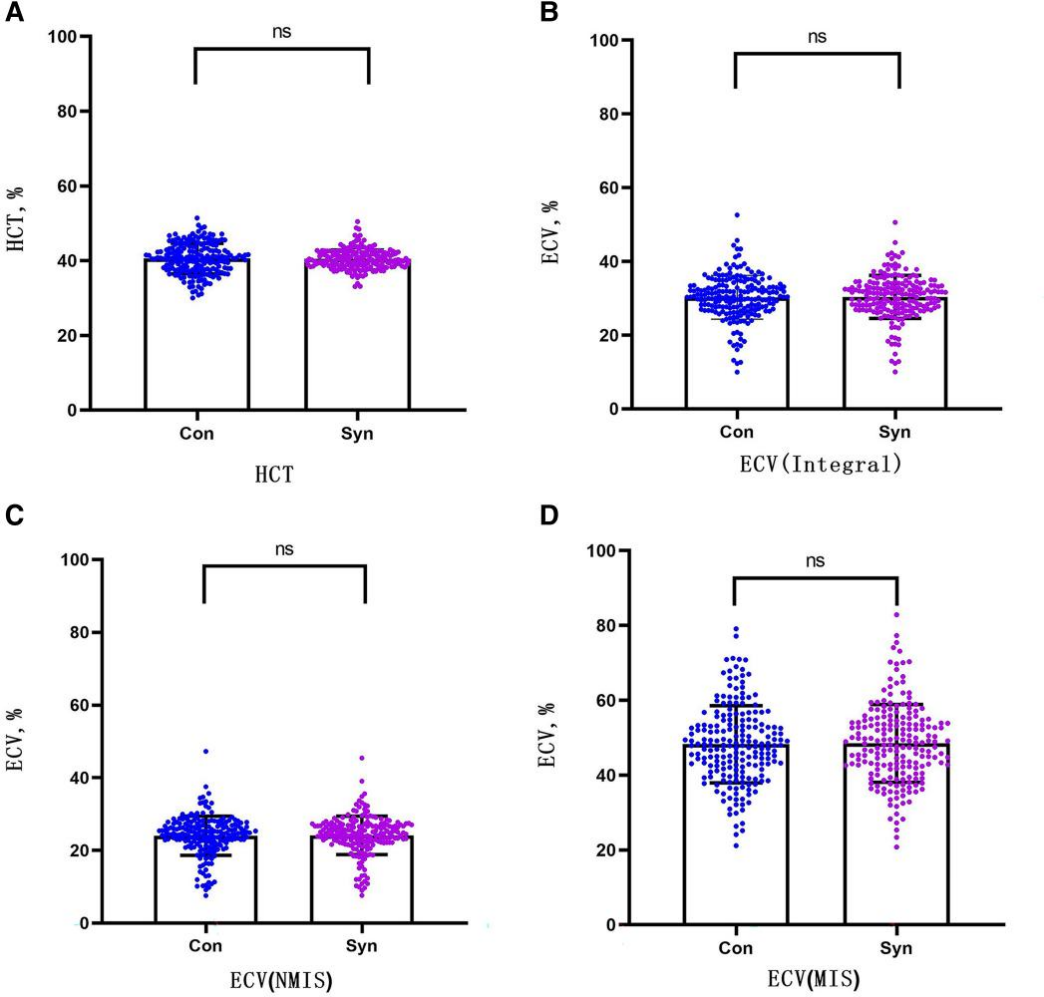
(*A*) Comparison between conventional HCT and synthetic HCT; (*B*) comparison between conventional ECV and synthetic ECV in the integral myocardium; (*C*) comparison between conventional ECV and synthetic ECV in NMIS; and (*D*) comparison between conventional ECV and synthetic ECV at MIS. NMIS, non-myocardial infarction sites; MIS, myocardial infarction site; ECV, extracellular volume; HCT, haematocrit; ns, not statistically significant.

**Figure 5:**
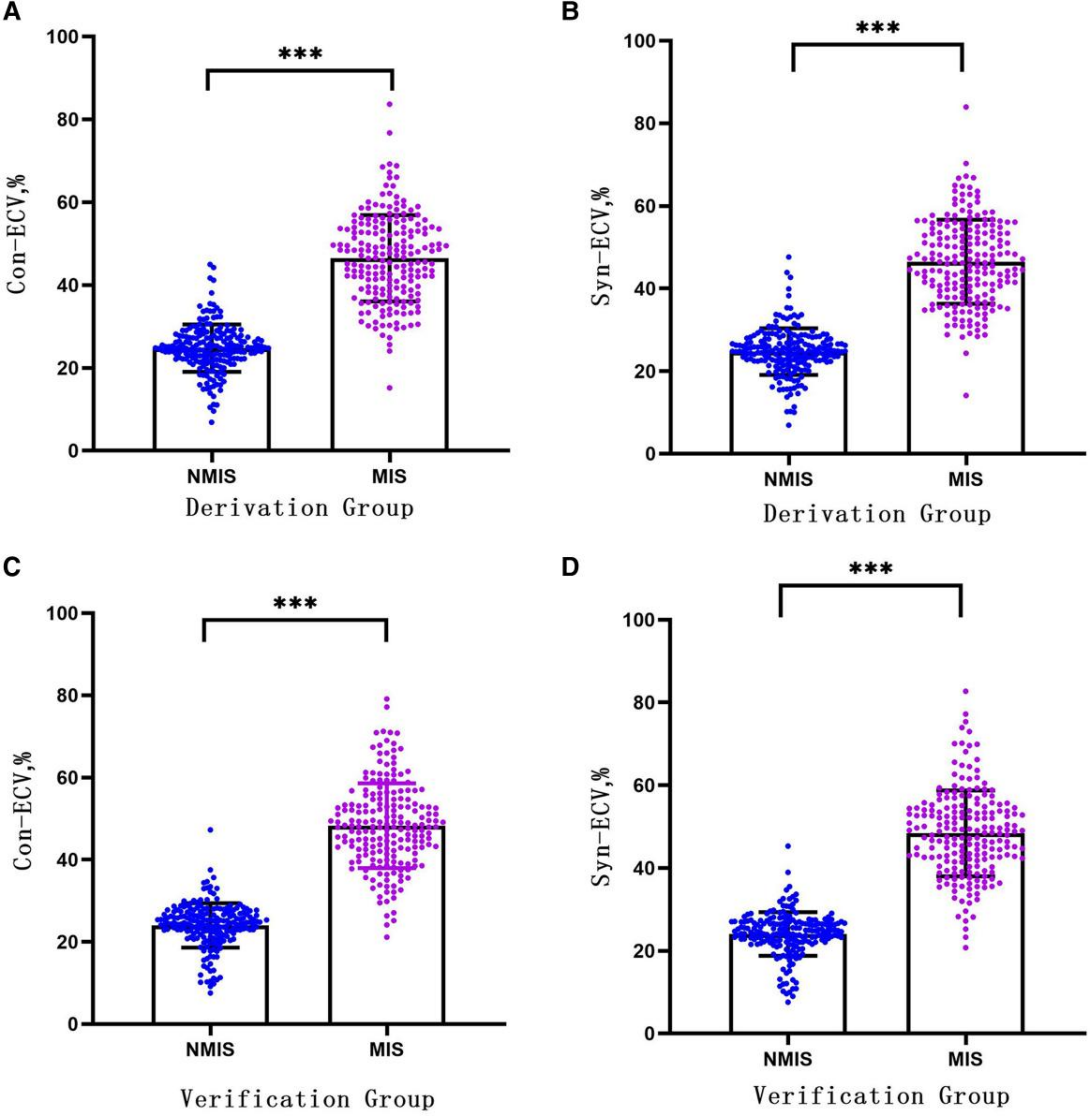
(*A*) Comparison of conventional ECV between NMIS and MIS in the derivation group; (*B*) comparison of synthetic ECV between NMIS and MIS in the derivation group; (*C*) comparison of conventional ECV between NMIS and MIS in the validation group; and (*D*) comparison of synthetic ECV between NMIS and MIS in the validation group. ***P < 0.001. NMIS, non-myocardial infarction sites; MIS, myocardial infarction site; ECV, extracellular volume; HCT, haematocrit.

In the verification group, the formula was used to calculate the synthetic HCT. Then, the synthetic HCT was used to calculate ECV. In the validation group, there was no statistical difference between conventional HCT and synthetic HCT (40.55 ± 4.09% vs. 40.48 ± 2.66%, *P* = 0.843). Also, there was no statistical difference between conventional ECV and synthetic ECV in the integral myocardium, MIS, and NMIS (30.26 ± 5.94% vs. 30.31 ± 5.81%, *P* = 0.925; 48.52 ± 10.33% vs. 48.38 ± 10.38%, *P* = 0.907; 24.02 ± 5.40% vs. 24.05 ± 5.27%, *P* = 0.952; *Figure 4* and *Table 3*). Similar to the conventional ECV, the synthetic ECV was significantly higher in MIS compared with that in NMIS (*P* < 0.001; *Figure 5*).

**Table 3:** Comparison of Pearson correlation coefficients of ECV

Validation (*n* = 199)	Conventional	Synthetic	*P*-value
HCT, %	40.55 ± 4.09	40.48 ± 2.66	0.843
ECV, % (integral)	30.26 ± 5.94	30.31 ± 5.81	0.925
ECV, % (NMIS)	24.02 ± 5.40	24.05 ± 5.27	0.952
ECV, % (MIS)	48.52 ± 10.33	48.38 ± 10.38	0.907

**Figure 6:**
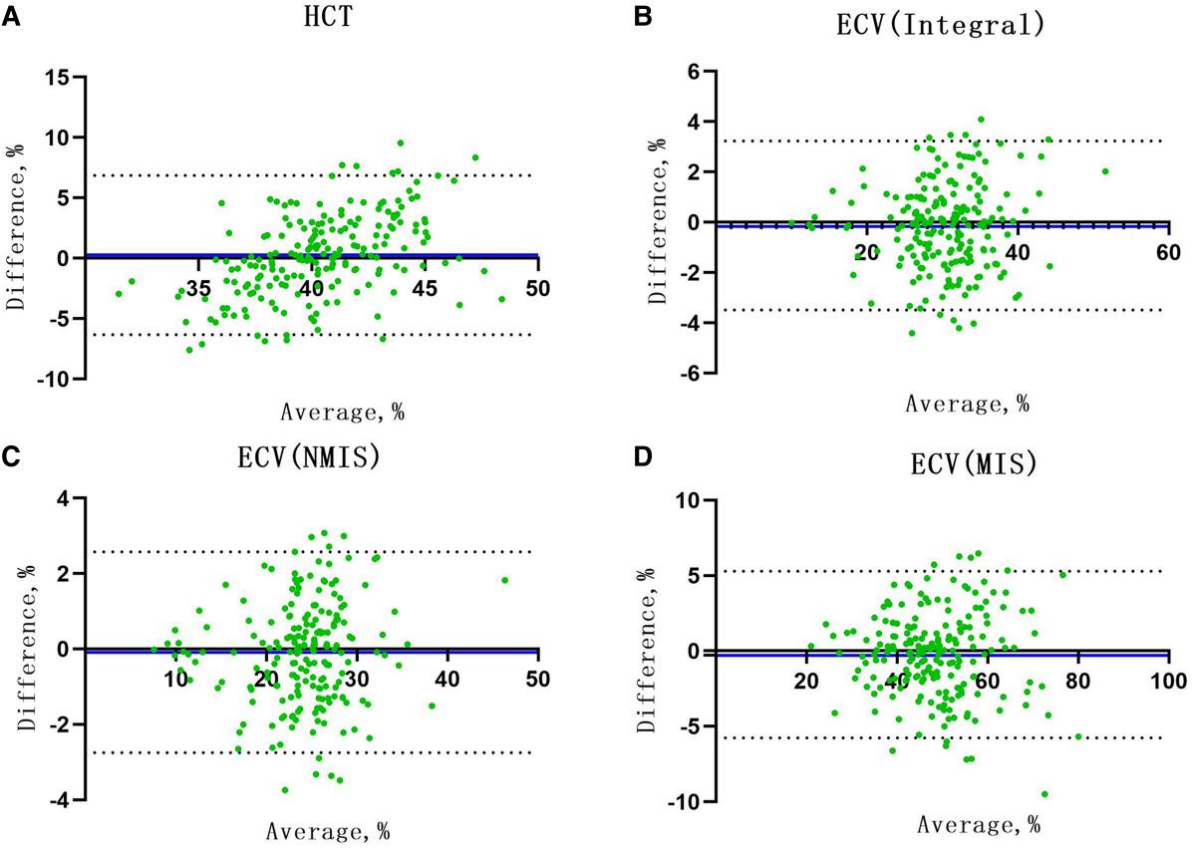
(*A*) Comparison between conventional HCT and synthetic HCT; (*B*) comparison between conventional ECV and synthetic ECV in the integral myocardium; (*C*) comparison between conventional ECV and synthetic ECV in NMIS; and (*D*) comparison between conventional ECV and synthetic ECV at MIS. The *X*-axis represents the mean value. The *Y*-axis represents bias. The dashed line is mean ± 1.96* SD. NMIS, non-myocardial infarction sites; MIS, myocardial infarction site; ECV, extracellular volume; HCT, haematocrit.

The Bland–Altman analysis showed good consistency between the conventional HCT and the synthetic HCT (bias = 0.28). The synthetic ECV and conventional ECV in integral, NMIS, and MIS also showed high consistency in the verification group (bias: −0.12, −0.09, and −0.23, respectively; *Figure 6*).

**Figure 7:**
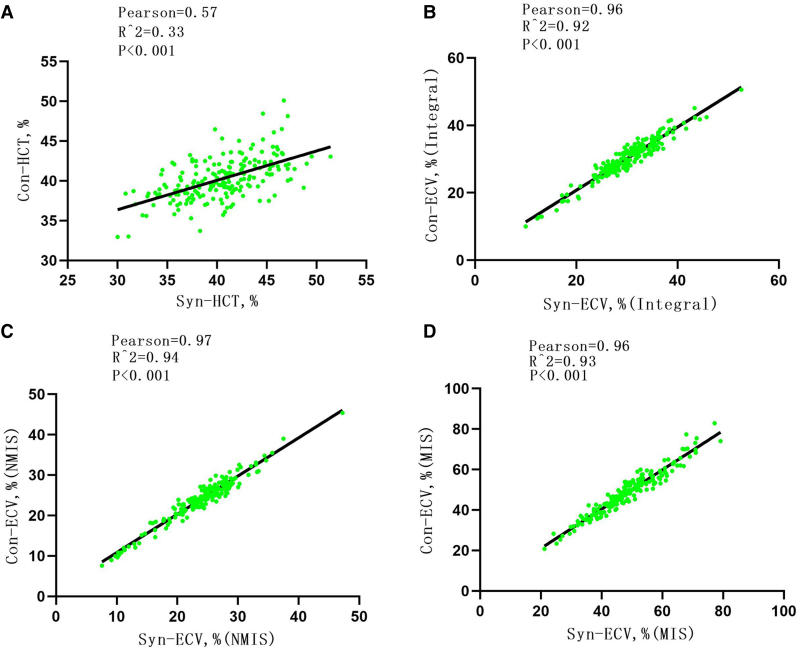
(*A*) Pearson analysis between synthetic HCT and conventional HCT; (*B*) Pearson analysis between synthetic ECV and conventional ECV in the integral myocardium; (*C*) Pearson analysis between synthetic ECV and conventional ECV in NMIS; and (*D*) Pearson analysis between synthetic ECV and conventional ECV in MIS. NMIS, non-myocardial infarction sites; MIS, myocardial infarction site; ECV, extracellular volume; HCT, haematocrit.

In addition, in the verification group, the conventional HCT and the synthetic HCT showed a linear correlation (*R*^2^ = 0.33, *P* < 0.001); the synthetic ECV and conventional ECV in the whole myocardium, MIS, and NMIS also showed a good linear correlation (*R*^2^ = 0.92, *P* < 0.001; *R*^2^ = 0.93, *P* < 0.001; *R*^2^ = 0.94, *P* < 0.001; *Figure 7*).

## Discussion

Previous studies have shown that synthetic ECV provided a new method for assessing myocardial fibrosis in patients with hypertrophic cardiomyopathy, aortic stenosis, and myocardial amyloidosis without blood sampling.^17–19^ However, there are currently no relevant data in the STEMI cohort to demonstrate the consistency and correlation between synthetic and conventional ECV. To our knowledge, this was the first time an ECV model without blood sampling had been constructed and validated in a STEMI cohort. The results of this study were as follows: (i) synthetic ECV in the STEMI cohort was very consistent and correlated with conventional ECV, regardless of infarct or NMIS, and (ii) the ECV of the MIS was significantly higher than that of the NMIS.

ECV was an essential tool for the assessment of myocardial fibrosis, known as ‘non-invasive’ or ‘virtual biopsy’, and was closely associated with poor prognosis after myocardial infarction.^11,20^ In our study, we found that both conventional and synthetic ECVs correlated with high-sensitivity troponin T in the MIS and integral, although this correlation was weak (see Supplementary data online, *Figure S1*). Therefore, ECV is valuable for the clinical management of STEMI patients. Using the linear relationship between HCT and magnetic resonance blood pool R1, formulas were derived to obtain synthetic HCT and ECV without blood sampling.^14,21–24^ Synthetic ECV may largely overcome the problem of conventional ECV requiring blood sampling, which makes it difficult to promote in the clinic. In this study, we found high concordance and correlation between conventional and synthetic ECV in the validation group, which provides some evidence for acquiring ECV without blood sampling in STEMI patients in the clinical setting. In the correlation analysis, we found that synthetic HCT and conventional HCT had moderate correlations, which may be related to the following reasons. First, the variability present in HCT itself affects the correlation between conventional and synthetic HCT. Treibel *et al.*^25^ measured HCT in 44 patients at 2 different times and found that the difference between measurements was as high as 10% (*R*^2^ = 0.86). However, due to the small sample size, more validation is needed for the generalizability of this finding. Secondly, factors that affect T1 relaxation times may also influence this relationship. Common influences on T1 relaxation times included temperature, haemoglobin oxygenation, and device parameters.^26–28^ Thirdly, HCT is usually measured in peripheral venous blood, whereas synthetic HCT is derived from the T1 relaxation time of arterial blood in the LV cavity. In fact, T1 blood measured in the ventricular cavity might be more accurate and precise than peripheral venous sampling,^29^ which explains why synthetic ECV performance seems to be better than it should be in cases where T1 is moderately correlated with peripheral blood HCT (*R*^2^ = 0.45). The high correlation between conventional and synthetic ECVs may be due to corrections for indicators other than HCT in the formulae.^30,31^ In addition, the present study found a high concordance and correlation between conventional and synthetic ECV, both in infarcted and non-infarcted regions. Synthetic ECV was significantly higher in the infarcted region compared with the non-infarcted region, suggesting more myocardial fibrosis. Therefore, synthetic ECV may be a suitable biomarker to differentiate between non-infarcted and infarcted myocardial sites.

Almost all published studies confirm that synthetic ECV obtained by synthetic HCT was feasible and that synthetic ECV was closely related to conventional ECV. The limited histological data also suggested that conventional and synthetic HCT were diagnostically equivalent. However, these above conclusions still lack further confirmation by studies with large sample sizes, which is what we are looking forward to in the future, as it may significantly enhance the clinical value of ECV.

### Limitations

First, this is a single-centre study, and because of differences in scanning parameters, equipment, and ethnicity, it is possible that the ECV model in this study needs to be more generalizable to other centres. Similarly, this study included all STEMI patients, so the findings of this study may not apply to patients with other diseases. In addition, whether different age stages and severity of anaemia may have an effect is a point of interest that needs to be explored in further studies.

## Conclusion

In summary, this study demonstrated the reliability of the synthetic ECV in STEMI patients without blood sampling. Compared with conventional ECV, synthetic ECV might be a simple and convenient non-invasive indicator to assess myocardial infarction.

## Supplementary data

Supplementary data are available at *European Heart Journal - Imaging Methods and Practice* online.

## Consent

Not applicable.

## Supplementary Material

qyae053_Supplementary_Data

## Data Availability

The data sets generated and/or analysed during the current study are available by request from the correspondences.

## Lead author biography

Wensu Chen, MD, PhD, Associate Chief Physician, Department of Cardiology, Affiliated Hospital of Xuzhou Medical University, Xuzhou, China, is the recipient of the 42nd American Heart Rhythm Society (AHRS)/Chinese Medical Association (CMA) Young Investigator Award (YIA) in 2021. She is a visiting scholar at the University of Chicago Medical Center, USA, and a visiting researcher at the Heart Center Berlin, Germany. She is mainly engaged in the field of cardiac magnetic resonance. She has published a number of papers in the field of cardiac magnetic resonance.

Wenliang Che, MD, PhD, Chief Physician, Tongji University School of Medicine, Shanghai Tenth People’s Hospital, Shanghai, China. Member of Atherosclerosis and Coronary Heart Disease Group of the 10th and 11th Committees of the Cardiovascular Society of the Chinese Medical Association, Young Member of Chest Pain Branch of the International Association for the Promotion of Medical Care, Professional Member of Shanghai Chest Pain Center Alliance, China Member of Cardiology Branch of Medical Association, Instructor of Coronary Intervention in China Cardiovascular Intervention Training Project, Assessment Expert of National Natural Science Foundation of China and Shanghai Natural Science Foundation, Examiner of Shanghai Resident Standardized Training Completion Examination. He has published >30 SCI papers as first author or corresponding author.

Lei Chen, MD, Tongji University, Shanghai, China, is mainly engaged in acute myocardial infarction and cardiac magnetic resonance–related research and has published >10 SCI papers as the first author.

## References

[1] Puymirat E, Simon T, Steg PG, Schiele F, Guéret P, Blanchard D et al Association of changes in clinical characteristics and management with improvement in survival among patients with ST-elevation myocardial infarction. JAMA 2012;308:998–1006. 22928184 10.1001/2012.jama.11348

[2] Gale CP, Allan V, Cattle BA, Hall AS, West RM, Timmis A et al Trends in hospital treatments, including revascularisation, following acute myocardial infarction, 2003–2010: a multilevel and relative survival analysis for the National Institute for Cardiovascular Outcomes Research (NICOR). Heart 2014;100:582–9. 24436220 10.1136/heartjnl-2013-304517

[3] Townsend N, Wilson L, Bhatnagar P, Wickramasinghe K, Rayner M, Nichols M. Cardiovascular disease in Europe: epidemiological update 2016. Eur Heart J 2016;37:3232–45. 27523477 10.1093/eurheartj/ehw334 PMC13480964

[4] Kristensen SD, Laut KG, Fajadet J, Kaifoszova Z, Kala P, Di Mario C et al Reperfusion therapy for ST elevation acute myocardial infarction 2010/2011: current status in 37 ESC countries. Eur Heart J 2014;35:1957–70. 24419804 10.1093/eurheartj/eht529

[5] Ibanez B, James S, Agewall S, Antunes MJ, Bucciarelli-Ducci C, Bueno H et al 2017 ESC guidelines for the management of acute myocardial infarction in patients presenting with ST-segment elevation: the Task Force for the management of acute myocardial infarction in patients presenting with ST-segment elevation of the European Society of Cardiology (ESC). Eur Heart J 2018;39:119–77. 28886621 10.1093/eurheartj/ehx393

[6] Taylor AJ, Salerno M, Dharmakumar R, Jerosch-Herold M. T1 mapping: basic techniques and clinical applications. JACC Cardiovasc Imaging 2016;9:67–81. 26762877 10.1016/j.jcmg.2015.11.005

[7] Garg P, Saunders LC, Swift AJ, Wild JM, Plein S. Role of cardiac T1 mapping and extracellular volume in the assessment of myocardial infarction. Anatol J Cardiol 2018;19:404–11. 29638222 10.14744/AnatolJCardiol.2018.39586 PMC5998858

[8] Robinson AA, Chow K, Salerno M. Myocardial T1 and ECV measurement: underlying concepts and technical considerations. JACC Cardiovasc Imaging 2019;12(11 Pt 2):2332–44. 31542529 10.1016/j.jcmg.2019.06.031 PMC7008718

[9] Banypersad SM, Fontana M, Maestrini V, Sado DM, Captur G, Petrie A et al T1 mapping and survival in systemic light-chain amyloidosis. Eur Heart J 2015;36:244–51. 25411195 10.1093/eurheartj/ehu444 PMC4301598

[10] De Meester De Ravenstein C, Bouzin C, Lazam S, Boulif J, Amzulescu M, Melchior J et al Histological validation of measurement of diffuse interstitial myocardial fibrosis by myocardial extravascular volume fraction from Modified Look-Locker imaging (MOLLI) T1 mapping at 3 T. J Cardiovasc Magn Reson 2015;17:48. 26062931 10.1186/s12968-015-0150-0 PMC4464705

[11] Chen BH, An DA, He J, Xu J-R, Wu L-M, Pu J. Myocardial extracellular volume fraction allows differentiation of reversible versus irreversible myocardial damage and prediction of adverse left ventricular remodeling of ST-elevation myocardial infarction. J Magn Reson Imaging 2020;52:476–87. 31943526 10.1002/jmri.27047

[12] Scully PR, Bastarrika G, Moon JC, Treibel TA. Myocardial extracellular volume quantification by cardiovascular magnetic resonance and computed tomography. Curr Cardiol Rep 2018;20:15. 29511861 10.1007/s11886-018-0961-3 PMC5840231

[13] Messroghli DR, Moon JC, Ferreira VM, Grosse-Wortmann L, He T, Kellman P et al Clinical recommendations for cardiovascular magnetic resonance mapping of T1, T2, T2* and extracellular volume: a consensus statement by the Society for Cardiovascular Magnetic Resonance (SCMR) endorsed by the European Association for Cardiovascular Imaging (EACVI). J Cardiovasc Magn Reson 2017;19:75. 28992817 10.1186/s12968-017-0389-8 PMC5633041

[14] Spees WM, Yablonskiy DA, Oswood MC, Ackerman JJ. Water proton MR properties of human blood at 1.5 Tesla: magnetic susceptibility, T1, T2, T*2, and non-Lorentzian signal behavior. Magn Reson Med 2001;45:533–42. 11283978 10.1002/mrm.1072

[15] Flett AS, Hayward MP, Ashworth MT, Hansen MS, Taylor AM, Elliott PM et al Equilibrium contrast cardiovascular magnetic resonance for the measurement of diffuse myocardial fibrosis: preliminary validation in humans. Circulation 2010;122:138–44. 20585010 10.1161/CIRCULATIONAHA.109.930636

[16] Thygesen K, Alpert JS, Jaffe AS, Chaitman BR, Bax JJ, Morrow DA et al Fourth universal definition of myocardial infarction (2018). Circulation 2018;138:e618–51. 30571511 10.1161/CIR.0000000000000617

[17] Censi S, Cimaglia P, Barbieri A, Naldi M, Ruggerini S, Brogneri S et al Performance of synthetic extracellular volume fraction in different cardiac phenotypes from a prospective cohort of patients referred for cardiac magnetic resonance. J Magn Reson Imaging 2021;54:429–39. 33590584 10.1002/jmri.27556

[18] Mesropyan N, Kupczyk P, Isaak A, Endler C, Faron A, Dold L et al Synthetic extracellular volume fraction without hematocrit sampling for hepatic applications. Abdom Radiol (NY) 2021;46:4637–46. 34109447 10.1007/s00261-021-03140-6 PMC8435519

[19] Chen W, Doeblin P, Al-Tabatabaee S, Klingel K, Tanacli R, Jakob Weiß K et al Synthetic extracellular volume in cardiac magnetic resonance without blood sampling: a reliable tool to replace conventional extracellular volume. Circ Cardiovasc Imaging 2022;15:e013745. 35360924 10.1161/CIRCIMAGING.121.013745 PMC9015035

[20] Kramer CM, Chandrashekhar Y, Narula J. T1 mapping by CMR in cardiomyopathy: a noninvasive myocardial biopsy? JACC: Cardiovascular Imaging 2013;6:532–4. 23579019 10.1016/j.jcmg.2013.02.002

[21] Piechnik SK, Ferreira VM, Lewandowski AJ, Ntusi NA, Banerjee R, Holloway C et al Normal variation of magnetic resonance T1 relaxation times in the human population at 1.5 T using ShMOLLI. J Cardiovasc Magn Reson 2013;15:13. 23331520 10.1186/1532-429X-15-13 PMC3610210

[22] Lu H, Clingman C, Golay X, van Zijl PCM. Determining the longitudinal relaxation time (T1) of blood at 3.0 Tesla. Magn Reson Med 2004;52:679–82. 15334591 10.1002/mrm.20178

[23] Shimada K, Nagasaka T, Shidahara M, Machida Y, Tamura H. In vivo measurement of longitudinal relaxation time of human blood by inversion-recovery fast gradient-echo MR imaging at 3T. Magn Reson Med Sci 2012;11:265–71. 23269013 10.2463/mrms.11.265

[24] Li W, Grgac K, Huang A, Yadav N, Qin Q, van Zijl PCM. Quantitative theory for the longitudinal relaxation time of blood water. Magn Reson Med 2016;76:270–81. 26285144 10.1002/mrm.25875 PMC4758918

[25] Treibel TA, Fontana M, Maestrini V, Castelletti S, Rosmini S, Simpson J et al Automatic measurement of the myocardial interstitium: synthetic extracellular volume quantification without hematocrit sampling. JACC Cardiovasc Imaging 2016;9:54–63. 26762875 10.1016/j.jcmg.2015.11.008

[26] Chow K, Flewitt JA, Green JD, Pagano JJ, Friedrich MG, Thompson RB et al Saturation recovery single-shot acquisition (SASHA) for myocardial T1 mapping. Magn Reson Med 2014;71:2082–95. 23881866 10.1002/mrm.24878

[27] Raucci FJ Jr, Parra DA, Christensen JT, Hernandez LE, Markham LW, Xu M et al Synthetic hematocrit derived from the longitudinal relaxation of blood can lead to clinically significant errors in the measurement of extracellular volume fraction in pediatric and young adult patients. J Cardiovasc Magn Reson 2017;19:58. 28768519 10.1186/s12968-017-0377-z PMC5541652

[28] Kellman P, Arai AE, Xue H. T1 and extracellular volume mapping in the heart: estimation of error maps and the influence of noise on precision. J Cardiovasc Magn Reson 2013;15:56. 23800276 10.1186/1532-429X-15-56 PMC3702513

[29] Wong TC, Piehler KM, Kang IA, Kadakkal A, Kellman P, Schwartzman DS et al Myocardial extracellular volume fraction quantified by cardiovascular magnetic resonance is increased in diabetes and associated with mortality and incident heart failure admission. Eur Heart J 2014;35:657–64. 23756336 10.1093/eurheartj/eht193 PMC3945798

[30] Puntmann VO, Carr-White G, Jabbour A, Yu C-Y, Gebker R, Kelle S et al Native T1 and ECV of noninfarcted myocardium and outcome in patients with coronary artery disease. J Am Coll Cardiol 2018;71:766–78. 29447739 10.1016/j.jacc.2017.12.020

[31] Nasser SB, Doeblin P, Doltra A, Schnackenburg B, Wassilew K, Berger A et al Cardiac myxomas show elevated native T1, T2 relaxation time and ECV on parametric CMR. Front Cardiovasc Med 2020;7:602137. 33330663 10.3389/fcvm.2020.602137 PMC7710854

