# Supplementary figures and images for: Establishment and validation of an extracellular volume model without blood sampling in ST-segment elevation myocardial infarction patients
Source: Eur Heart J Imaging Methods Pract. 2024 Jun 10;2(1):qyae053. doi: 10.1093/ehjimp/qyae053 (PMC11367959; doi:10.1093/ehjimp/qyae053)

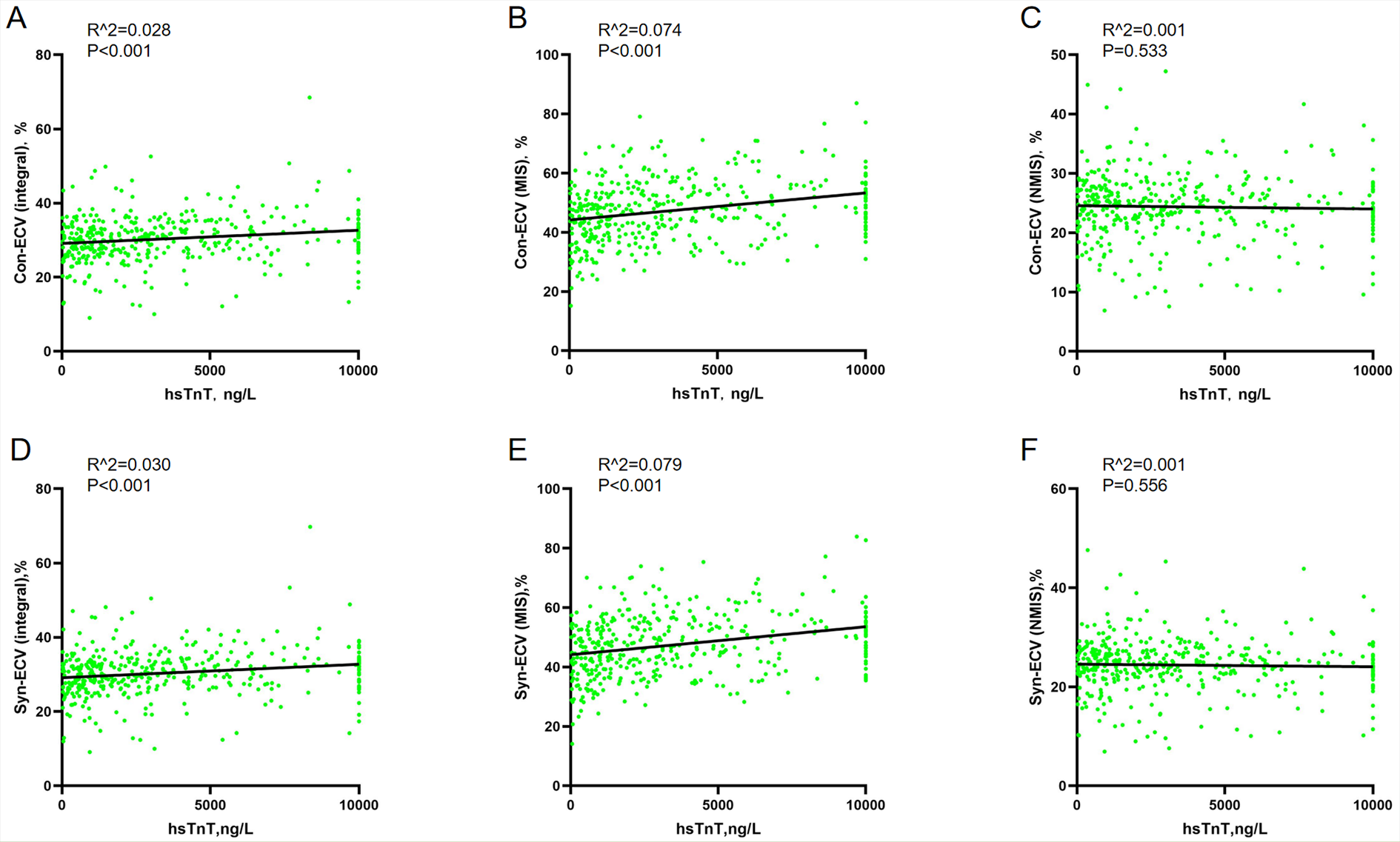

Supplement: qyae053_Supplementary_Data [file qyae053_Supplementary_Data.tif]
